# Co-circulation of *Chikungunya virus*, *Zika virus*, and serotype 1 of *Dengue virus* in Western Bahia, Brazil

**DOI:** 10.3389/fmicb.2023.1240860

**Published:** 2023-08-23

**Authors:** Marcus Vinicius de França Cirilo, Shahab Zaki Pour, Viviane de Fatima Benedetti, Jéssica Pires Farias, Mayanna Moreira Costa Fogaça, Rafael da Conceição Simões, Paloma Oliveira Vidal, Alexander Birbrair, Paolo Marinho de Andrade Zanotto, Wilson Barros Luiz, Jaime Henrique Amorim

**Affiliations:** ^1^Western Bahia Virology Institute, Center of Biological Sciences and Health, Federal University of Western Bahia, Barreiras, Brazil; ^2^Laboratory of Molecular Evolution and Bioinformatics, Department of Microbiology, University of Sao Paulo, Sao Paulo, Brazil; ^3^Laboratory of Biochemistry and Plant Physiology, Center of Biological Sciences and Health, Federal University of Western Bahia, Barreiras, Brazil; ^4^Department of Dermatology, School of Medicine and Public Health, University of Wisconsin-Madison, Madison, WI, United States; ^5^Laboratory of Applied Pathology and Genetics, Department of Biological Sciences, State University of Santa Cruz, Ilhéus, Brazil

**Keywords:** RT-PCR, arbovirus, co-circulation, Western Bahia, phylogeny

## Abstract

Chikungunya, mayaro, dengue, zika, and yellow fever are mosquito-borne viral diseases caused, respectively, by *Chikungunya virus*, *Mayaro virus* (CHIKV and MAYV, respectively: *Togaviridae*: *Alphavirus*), *Dengue virus*, *Zika virus, and Yellow fever virus* (DENV, ZIKV, and YFV, respectively: *Flaviviridae*: *Flavivirus*). These viruses have an important epidemiological impact worldwide, especially in Brazil. Western Bahia is one of the less studied regions in that country regarding the circulation of these pathogens. In this study, we aimed to apply molecular biology assays to better know the mosquito-borne viruses circulating in Barreiras and Luís Eduardo Magalhães, two main cities of Western Bahia. From March to June 2021, we enrolled 98 patients with the clinical diagnosis of dengue. Personal information (gender and age) were retrieved at the moment of enrollment. Serum samples were obtained from volunteers and used in molecular detection of CHIKV, MAYV, DENV, ZIKV, and YFV by reverse transcription followed by real-time polymerase chain reaction as well as in genome sequencing aiming phylogenetic analysis. As the main result, we found that from the 98 patients 45 were infected by CHIKV, 32 were infected by serotype 1 of DENV (DENV-1) and six were infected by ZIKV, while 15 were negative for all arboviruses tested. In addition, phylogenetic analysis revealed that all CHIKV-positive samples were of the East/Central/South African (ECSA) genotype, while all DENV-1-positive samples were of the V genotype. These results clearly show that epidemiological surveillance cannot be based only on clinical evaluations. Laboratory diagnosis is important in arbovirus infection that are prevalent in a particular area. These findings also demonstrate the co-circulation of many arboviruses in Western Bahia in 2021.

## Introduction

Chikungunya is a mosquito-borne disease caused by the *Chikungunya virus* (CHIKV: *Togaviridae*: *Alphavirus*; Weaver and Lecuit, 2015). CHIKV genome is based on a single-stranded, positive-sense RNA of ~12 kb, which contains two open reading frames (ORFs) encoding non-structural (nsP1, nsP2, nsP3, and nsP4) and structural proteins (C, E3, E2, 6K, and E1; Strauss and Strauss, 1994). CHIKV was first described during a “dengue-like” outbreak in Tanzania in the beginning of the 1950’s (Lumsden, 1955). The disease caused by this virus is characterized by fever, asthenia, arthralgia, myalgia, headache and rash (Weaver and Lecuit, 2015). CHIKV infection was first reported in Brazil in 2010, based on serological tests (De Albuquerque et al., 2012). Two years later, molecular analyses revealed its simultaneous introduction in two Brazilian regions: Northern, in Amazonas state (Asian genotype virus) and Northeast (East/Central/South African ECSA genotype), in two cities: Feira de Santana and Riachão do Jacuípe, both in Bahia state (Nunes et al., 2015; Teixeira et al., 2015). The number of chikungunya cases confirmed by clinical criteria from 2014 to 2020 were 2,404; 14,995; 126,256; 111,974; 47,737; 69,804, and 21,763, respectively (Cidades - Plataforma Integrada de Vigilância em Saúde - Ministério da Saúde, 2022). The disease has affected more than 55% of the Brazilian municipalities. Bahia is the most CHIKV-affected Brazilian state.

Dengue and zika are also mosquito-borne diseases caused by *Dengue virus* and *Zika virus*, respectively (DENV and ZIKV, respectively: *Flaviviridae*: *Flavivirus*; Lindenbach et al., 2007). DENV has the highest medical and epidemiological importance in the *Flavivirus* genus. It is responsible for more than 390 million annual infections worldwide by one of its four serotypes (DENV- 1-4; Bhatt et al., 2013). All of the DENV serotypes have a great epidemiological impact in Brazil, with more than 7 million cases of dengue notified from 2011 to 2020 (Cidades - Plataforma Integrada de Vigilância em Saúde - Ministério da Saúde, 2022). ZIKV was previously restricted to circulating in African and Asian countries, causing mild infectious. However, it was introduced in the Americas and was associated with microcephaly in newborns and Guillain-Barré syndrome in adults, mainly in Brazil, in which its first outbreak was reported in 2015, in Bahia state (Campos et al., 2015; Areas with Zika | Zika virus | CDC, 2016; Calvet et al., 2016; Wikan and Smith, 2016). The genomes of DENV and ZIKV are consist of a single-stranded positive RNA which is protected by an icosahedral capsid and an envelope. The RNA is translated shortly after the virus entering the host cell. It encodes three structural proteins: capsid protein (C), pre-membrane protein (pre-M) and the envelope glycoprotein (E). In addition, the genomic RNA also encodes seven non-structural (NS) proteins: NS1; NS2A; NS2B; NS3; NS4A, NS4B and NS5 (Lindenbach et al., 2007; Guzman et al., 2016).

**Figure 1:**
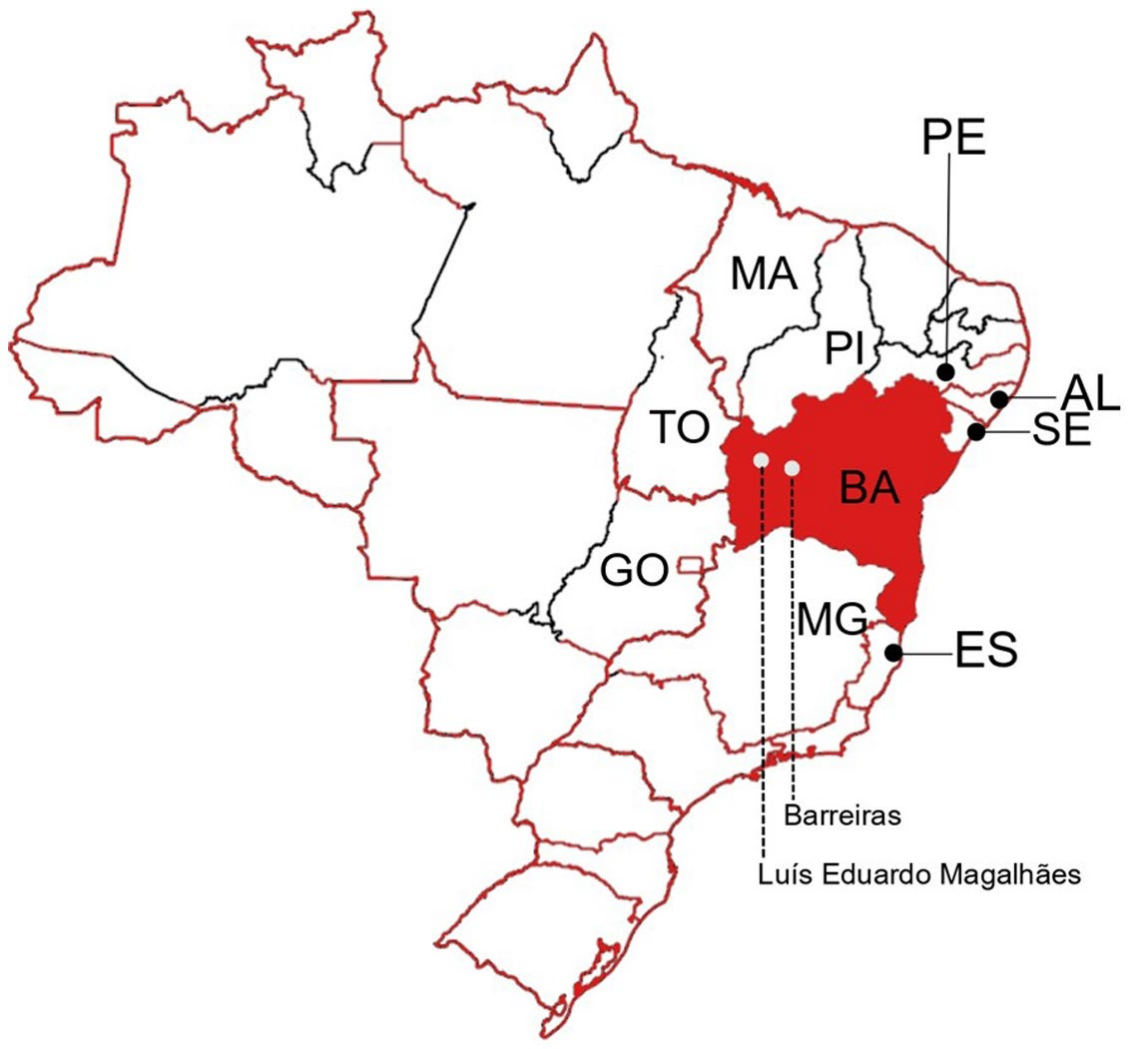
Representation of the Brazilian territory as a map. Borders of Brazilian states are shown. Bahia (BA) state is highlighted in red. The neighbor states of Sergipe (SE), Alagoas (AL), Pernambuco (PE), Piauí (PI), Maranhão (MA), Tocantins (TO), Goiás (GO), Minas Gerais (MG), and Espírito Santo (ES) are indicated. Barreiras and Luís Eduardo Magalhães cities are indicated in the Western Bahia.

The Western Bahia is one of the less studied regions of the state regarding arboviral diseases As far as we are aware, investigations that detail the specific arboviruses that have historically circulated in the area have not been published. Barreiras is the main city of the Western Bahia region, with an estimated population of 158,432 inhabitants (IBGE | Cidades@ | Bahia | Barreiras | Panorama, 2021). Luís Eduardo Magalhães (LEM) is a neighbor city of Barreiras, with an estimated population of 92,671 inhabitants (IBGE | Cidades@ | Bahia | Luís Eduardo Magalhães | Panorama, 2022; see Figure 1). According to the Brazilian Ministry of Health, 3,130, 61 and 247 cases of dengue, chikungunya and zika, respectively, were reported from 2017 to 2020 in Barreiras city. In addition, 1,417, 4 and 3 cases of dengue, chikungunya and zika, respectively, were reported from 2017 to 2020 in LEM (Cidades - Plataforma Integrada de Vigilância em Saúde - Ministério da Saúde, 2022). Most of reports are based only on clinical criteria. A minor proportion of them are based on serologic laboratory assays. In this study, we aimed to apply molecular biology assays to better know the arboviruses circulating in Barreiras and LEM.

## Materials and methods

### Ethics approval and study design

All the research complied with all relevant ethical and biosafety guidelines. Ethics approval was obtained from institutional ethics committee of the Federal University of Western Bahia (CAAE: 42901721.9.0000.8060) and also by the Institute of Biomedical Science of the University of São Paulo (ICB-USP) Ethical Committee (process number of CEPSH-ICB 3.558.917). All procedures and possible risks were explained to volunteers. Informed consent was obtained from all participants. Research was performed in accordance with relevant guidelines/regulations. From March to June 2021, patients with clinical diagnosis of dengue in Barreiras and Luís Eduardo Magalhães (LEM) cities, Bahia, Brazil were invited to be volunteers in this study. We enrolled 98 patients (19 from Barreiras and 81 from LEM) that agreed to donate a blood sample in the acute phase of the disease, up to the fifth day after the symptom’s onset. The age of the participants ranged from 1 to 65 years old, and the median was 31 years old. Fifty-two (53.06%) female subjects and 48 (46.94%) male subjects composed the group of participants. Personal information (gender and age) were retrieved at the moment of enrollment. Detailed demographic information are given in Supplementary material 1. Serum samples were obtained from the blood samples after low centrifugation, as previously described (Farias et al., 2023), and kept at −80°C until analyses.

### RNA extraction and RT-PCR

Viral RNA was extracted using the automated Extracta 32 system (Loccus, Brazil), as recommended by the manufacturer. *Zika virus* (ZIKV), *Chikungunya virus* (CHIKV), and *Dengue virus* (DENV) serotypes 1–4 were detected by one-step reverse transcription followed by real-time polymerase chain reaction (RT-PCR) using the ZDC Biomol kit (CN:210085Z001 IBMP, Brazil), as recommended by the manufacturer. Alternatively, two-step RT-PCR assays were carried out using primers and probes specifically designed to detect *Zika virus* (ZIKV; Lanciotti et al., 2008), *Chikungunya virus* (CHIKV; Patel et al., 2016), *Yellow fever virus* (YFV; Diallo et al., 2014) and *Mayaro virus* (MAYV; Long et al., 2011). Then, the samples with a Ct value of less than 29 were included in the sequencing procedure.

### Oligonucleotide primers for DENV and CHIKV

The 43 available complete DENV-1 sequences (Envelope protein-E) were retrieved from NCBI (National Center for Biotechnology Information) available from https://www.ncbi.nlm.nih.gov by January, 2022 (Supplementary material 2). The sequences were aligned with Multiple Alignment using Fast Fourier Transform- MAFFT (v7.453–1; Katoh et al., 2002). Subsequently, the alignment was submitted to the Primerdesign-M available in the Los Alamos HIV webtool (Yoon and Leitner, 2015). From more than 20 primer sets suggested, 4 pairs of primers were chosen considering the Blast result, amplicon size between 700 to 800 base pairs, overlap size, and the difference of 5°C in the melting temperature for each pair of the primers (see Table 1). For CHIKV we used primers 21 and 22 targeting the E1–3’UTR region, as previously described (Stapleford et al., 2016).

**Table 1:** Oligonucleotide primers used to amplify DENV-1 sequences.

Set	Primer Name	Sequence 5′-3′	Position	Annealing temperature	Amplicon (bp)
1	EF-1	TCTAGAAACAAGAACYGAAAC	648	55°C	676
	ER-1	GCTTGAGGTGTTATGGTTGC	1,324		
2	EF-2	AAGATGTCCAACACAAGGAG	1,056	55°C	687
	ER-2	GCTTAAATGAGCCTGTGCAC	1,743		
3	EF-3	ACCATAACACCTCAAGCTCC	1,327	55°C	700
	ER-3	TCGAACATTTTCCCTATGCTGC	2,027		
4	EF-4	GAGAAGGAAGTGGCTGAGAC	1,765	55°C	667
	ER-4	GACAGTCTTTTTGGGGAGTCAG	2,432		

### cDNA synthesis

Extracted RNA was converted to cDNA with High-Capacity cDNA Reverse Transcription Kits (Applied Biosystem) using random primers. Briefly, 20 μL reactions containing 5 μl of nucleic acids, 2 μl of 10 mM random primer, 1 μl multiScribe RT, 2.5 μl 10X RT buffer, 1 μl (0.1 M) DTT, 0.8 μl (10 mM) of each dNTPs, 1 μl (40 U/μl) RNaseOUT ribonuclease inhibitor (Invitrogen), and 6.7 μl nuclease-free water was incubated at 25°C for 10 min, 37°C for 120 min, with an inactivation step of 85°C for 5 min.

### Amplicon generation

Amplicons were generated in 22 μl reactions containing 2 μl ZIKV cDNA, 0.5 μl each (10 mM) forward and reverse primers, 0.1 μl PlatinumTaq DNA polymerase high Fidelity (Invitrogen), 2.5 μl 10X PCR buffer (reaching a final concentration of 1.13 x per reaction), 1 μl (50 mM) MgSO4, 0.5 μl (10 mM) dNTPs, and 14.9 μl nuclease-free water. The cycling conditions with MJ research PTC-200 Peltier thermal cyclers from bio-rad consisted of amplification was carried out as follows: 94°C for 5 min, followed by 40 cycles at 94°C for 15 s, 55°C for 30 s, and 68°C for 2 min and 30 s in the case of short amplicons and 4 min and 30 s in the case of long amplicons, followed by a final extension at 68°C for 10 min. Amplicons were visualized on 1% agarose gels submitted to electrophoresis at 90 volts for 90 min in TAE buffer 1%, utilizing the ladder of 100 bp (Invitrogen).

### Next-generation sequencing

Five dengue-positive samples and nine chikungunya-positive samples were sequenced, using next generation sequencing (NGS) on Oxford Nanopore’s MinIon platform. All ZIKV samples showed Ct values above 29 and we could not generate amplicons, so they were not subjected to sequencing (data not shown). Viral RNA was extracted as described above. Samples of extracted RNA were subjected to two-step reverse transcription followed by polymerase chain reaction, as described above. The amplicons were confirmed by 1% (w/v) agarose gel electrophoresis. End-prep reactions were performed with NEBNext® Ultra™ II End Repair/dA-Tailing Module (New England Biolab, USA) and barcoded using ONT Native Barcoding Expansion Kit (EXP-NBD104; Oxford Nanopore technologies, UK). The barcoded samples were then combined, purified with AMPure XP Beads (Backman Couter, USA) and loaded onto Oxford Nanopore MinION SpotON flow cells R9.4.1 (Oxford Nanopore Technologies, UK), prepared according to version 3 of the ARTIC nCoV-2019 sequencing protocol [nCoV-2019 sequencing protocol v3 (LoCost) (protocols.io)]. The sequencing was performed using the MinKNOW device, with mapping of the reads generated by the Basecalling fast accuracy method. ARTIC Network’s read assignment, mapping, and phylogenetic analysis in real time - RAMPART software^1^ was used to monitor the sequencing run in real-time to estimate the depth of coverage (20x) across the entire sequence for each barcode.^2^

### Next-generation sequencing data analysis

Raw data quality was checked with the FastQC v 0.11.6 program (Andrews, 2010). Then, sequencing data yielded from MinIon was filtered to remove low-quality reads with Qcat v1.1.0. Moreover, the same program was used to demultiplex and adapters trimming ONT barcoded reads and assign to the correct sample using default parameters.^3^ Then, reads quality score greater than seven was subjected to mapping to genome references (Accession number of KP188543 and, Accession number of KP164572 for DENV-1 and CHIKV respectively) performed by Minimap2 with the default parameters (Li, 2018, 2021). Consequently, the alignment was manipulated with SAMtools v 1.11 to create a sorted BAM file format (Li et al., 2009). Finally, and consensus sequence generated by Ugene v42.0 (Okonechnikov et al., 2012). Sequences generated in this study were deposited in GenBank of NCBI, and accession numbers are listed in the Supplementary material 3.

### Phylogenetic analysis

The phylogenetic trees were reconstructed based on the E protein (DENV-1) or E1–3’UTR region (CHIKV), using the Maximum Likelihood (ML) method implemented in IQ-TREE 1.5.5 [14]. The robustness of the groupings observed was assessed using an ultrafast bootstrap approximation (UFboot) during 1,000 replicates. The ML trees were visualized and plotted using FigTree v.1.4.3 [15].

## Results

### Molecular diagnosis

According to clinical evaluations, all of the 98 patients were reported as dengue cases. However, molecular diagnosis based on RT-PCR showed that we had 45 cases of chikungunya, 32 cases of dengue (all of them serotype 1) and six cases of zika. From the 98 patients enrolled in this study, 15 were negative for all arboviruses tested by our RT-PCR strategies: *Dengue virus*, *Zika virus*, *Yellow fever virus*, *Mayaro virus*, and *Chikungunya virus* (Table 2). These results clearly show that both, clinical diagnosis and epidemiological surveillance of arboviruses must involve laboratory investigation methods, especially those capable of detecting viral nucleic acid. In addition, these results show that there was a co-circulation of different arboviruses in Western Bahia in 2021. Demographic information of patients according to RT-PCR results are given in Supplementary material 4.

**Table 2:** Percentage of patients infected or non-infected by viruses, and by age group.

Age group (Years)	DENV-1	CHIKV	ZIKV	Non-Detected
1–12	15.30%	8.20%	1.02%	2.04%
13–24	5.10%	17.34%	3.06%	5.10%
25–36	7.14%	7.14%	1.02%	6.12%
37–48	2.04%	9.18%	1.02%	1.02%
49–60	2.04%	3.06%	-	1.02%
>60	1.02%	1.02%	-	-
TOTAL (100%)	32.64%	45.94%	6.12%	15.30%

### Phylogeny

**Figure 2:**
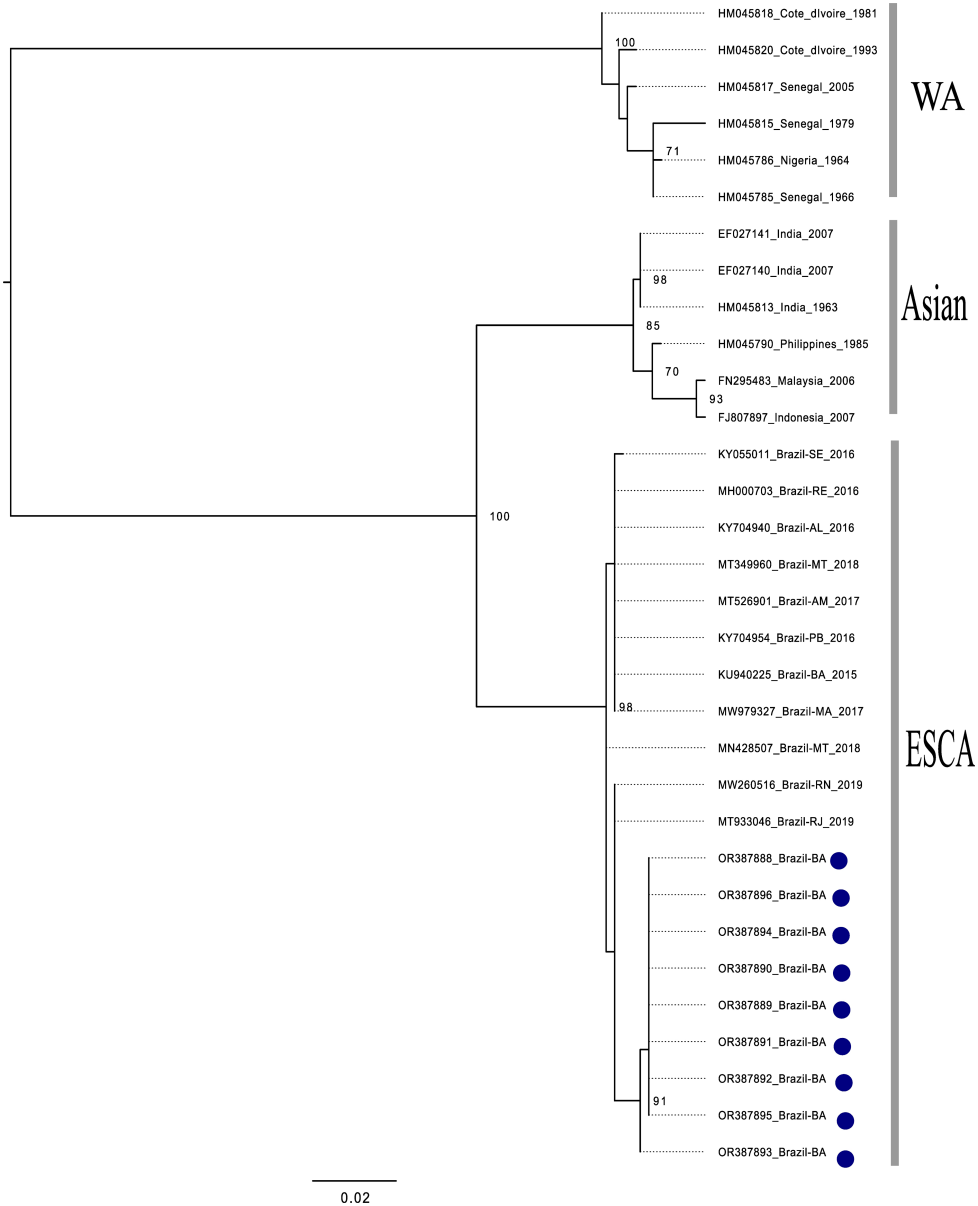
Phylogenetic analysis of CHIKV, based on partial sequences of the E1-3’UTR (untranslated) region. The phylogenetic tree was constructed using the maximum likelihood method with a bootstrap of 1,000 replicates. The dataset consisted of 32 CHIKV nucleotide sequences with: West African genotype (*n* = 06), Asian genotype (*n* = 06), ECSA genotype (*n* = 11) and the sequences generated in this study (*n* = 9). All sequences retrieved from NCBI are identified in the format: accession number/country/year of isolation. Sequences generated in this study are identified according to its internal number and country of isolation (blue dots). The bootstrap values are very similar to the isolates from southeastern Brazil, being grouped with the isolates from Rio de Janeiro. It is thus inferred that these isolates belong to the ECSA lineage, the isolates from Bahia (2020–2021) form a monophyletic group with high support value, indicating that they had the same evolutionary origin.

**Figure 3:**
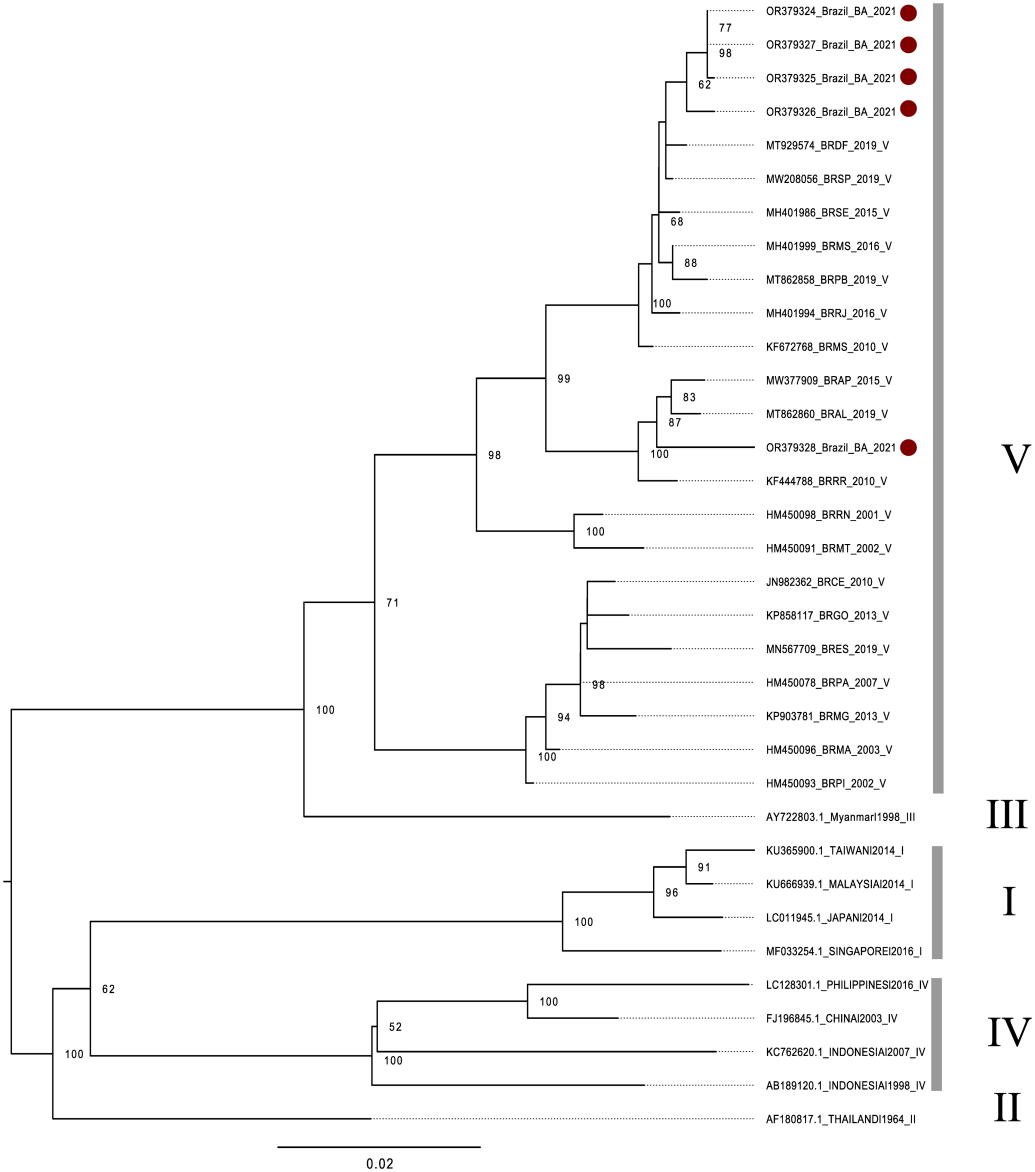
Phylogenetic analysis of DENV-1, based on a fragment of the E protein coding sequence. The phylogenetic tree was built using the maximum likelihood method with bootstrap of 1000 replicates. The five DENV-1 isolates generated in the study were aligned to 29 global reference sequences. All sequences retrieved from NCBI are identified in the format: accession number/ country/ year of isolation. Brazilian sequences: BR (Brazil), followed by the acronym of the Brazilian state. Regarding sequences generated in this study: four isolates (red dots, above) are closely related to other strains isolated in the Federal District (DF) and São Paulo (SP), in 2019. In addition, one isolate (red ball, below) is related to strains isolated in the state of Alagoas (AL), in 2019 and Amapá (AP), in 2015 (AP).

All of the nine CHIKV-positive samples could be used to generate amplicons suitable for sequencing. According to the phylogenetic analysis conducted with the generated reads, all of the CHIKV-positive samples were shown to be of the East/Central/South African (ECSA) genotype (Figure 2). In addition, all of the five DENV1-positive samples could be used to generate amplicons suitable for sequencing. According to the phylogenetic analysis conducted with the generated reads, all of the DENV1-positive samples were shown to be of the V genotype (Figure 3). Collectively, these results indicate that both, CHIKV and DENV1 detected in this study were genetically related with viruses previously introduced in Brazil.

## Discussion

Most of diseases caused by arthropod borne viruses (arboviruses) occurring worldwide are reported without a laboratory investigation (Lima-Camara, 2016; Donalisio et al., 2017). Among cases confirmed based on laboratory methods, the serological assays are the most frequently used. However, such analyses do not precisely distinguish arboviruses from the same genus (Donalisio et al., 2017; Piantadosi and Kanjilal, 2020). Such a differentiation can be achieved by using molecular biology analyses, such as the reverse transcription followed by real-time polymerase chain reaction (RT-PCR; Pabbaraju et al., 2016). Unfortunately, such molecular techniques are not used on a regular basis, especially in developing countries.

As an example, we showed in this study that from 98 patients diagnosed with dengue without a laboratory investigation, 45, 32 and six were actually infected by CHIKV, DENV-1, and ZIKV, respectively, while 15 of them were actually negative for all arboviruses tested: *Dengue virus*, *Zika virus*, *Yellow fever virus*, *Mayaro virus* and *Chikungunya virus*. Such a discrimination was only possible due to the use of RT-PCR to find the correct diagnosis. Detection of the viral nucleic acid is one of the more confident and reliable laboratory methods to diagnose infection by DENV and other arboviruses (WHO | Dengue guidelines for diagnosis, treatment, prevention and control: new edition, 2014). After the onset of illness, the virus can be detected in serum, plasma, circulating blood cells and other tissues for 4–5 days. Most of arboviruses, such as DENV and CHIKV cause identical or at least very similar clinical syndromes (Dong et al., 2012). Clinical diagnosis and epidemiological surveillance of arboviruses must involve laboratory investigation methods, especially those capable of detecting viral nucleic acid. In order to better understand arboviruses circulating in a given area, molecular diagnosis is essential.

In addition to the better precision in diagnosis, epidemiological surveillance of arboviruses can also benefit from the use of molecular biology techniques when gene sequencing approaches are used to understand or at least infer the origin of viruses circulating in a given region. In this study, we showed that all CHIKV that were circulating in the study area belongs to the ECSA genotype. Interestingly, CHIKV that were introduced in Bahia in 2014 were shown to belong to the same genotype. It is reasonable to think that these viruses descend from those originally introduced in the same state but in a different region. Feira de Santana, the main original outbreak focus of CHIKV is almost 800 Km distant from our study area. However, sequences generated in this study were shown to be closely related to isolates from Rio de Janeiro (RJ) and Rio Grande do Norte (RN) states. The same ECSA genotype of CHIKV is the most frequent detected in Brazil and nowadays is present in different regions of the country (Souza et al., 2019; de Lima et al., 2021; De Oliveira et al., 2021). ECSA genotype of CHIKV introduction in Western Bahia may have occurred years before the beginning of our surveillance detection.

Another important result of this study was that all of DENV-1-positive samples were shown to belong to the V genotype, according to the phylogenetic analysis. In Brazil, DENV-1 was introduced in the 80’s, and remained the prevalent serotype until its replacement by DENV-2 in the 90′s, which was subsequently replaced by DENV-3 in 2000 (Drumond et al., 2012). DENV-1 re-emerged only in 2009 after approximately eight years without being related to epidemics (de Bruycker-Nogueira et al., 2018). The co-circulation of different DENV-1 lineages was identified, however, its transmission dynamics afterwards, was not fully characterized. However, a continuous molecular surveillance after the reemergence period (2012 to 2016), covering the 30 years of circulation of DENV-1 in Brazil revealed a continued presence of genotype V, as well as three distinct co-circulating lineages (de Bruycker-Nogueira et al., 2018). As far as we know, at least a large majority of Brazilian isolates of DENV-1 belongs to genotype V (Ribeiro et al., 2021). Thus, our finding is in accordance with the historic presence of the genotype V of DENV-1 in Brazil.

The observation that the ECSA genotype of CHIKV was circulating undetected in Western Bahia underscores the need for improvements in molecular methods for viral surveillance. In the same way, the miss-diagnosis of DENV instead of ZIKV or CHIKV seen in this study reinforces the need for the use of RT-PCR in the diagnosis of arboviral diseases. Unfortunately, we were not able to sequence ZIKV-positive samples due to technical limitations related to high Ct values and difficulties in generating amplicons for sequencing. Nonetheless, the precise determination of DENV serotype and genotype was only possible due to the use of molecular methods. This study strengthens the relevance of molecular surveillance in order to identify, trace, and control arboviruses circulating in Brazil.

## Data availability statement

The datasets presented in this study can be found in online repositories. The names of the repository/repositories and accession number(s) can be found in the article/Supplementary material.

## Ethics statement

The studies involving humans were approved by all the research complied with all relevant ethical and biosafety guidelines. Ethics approval was obtained from institutional ethics committee of the Federal University of Western Bahia (CAAE: 42901721.9.0000.8060) and also by the Institute of Biomedical Science of the University of São Paulo (ICB-USP) Ethical Committee (process number of CEPSH-ICB 3.558.917). The studies were conducted in accordance with the local legislation and institutional requirements. Written informed consent for participation in this study was provided by the participants’ legal guardians/next of kin.

## Author contributions

MFr enrolled patients, collected samples, carried out RT-PCR, and analyzed the data generated. SP carried out RT-PCR, prepared cDNA samples, conducted phylogenetic analyses, and wrote the paper. VB carried out RT-PCR, and prepared cDNA samples. JF and MFo carried out RT-PCR, and analyzed the data generated. RC, PV, AB, PA, and WL analyzed the data, gave scientific support and wrote the paper. JA conceived the study, gave scientific support, analyzed the data, and wrote the paper. All authors contributed to the article and approved the submitted version.

## Funding

This research was funded by Financiadora de Estudos e Projetos (FINEP), project 29334 -FINEP/UFMG/REITORIA/PRPQ/LABORATÓRIOS DE CAMPANHA FASE II -Sub: 06 (JA); FINEP 1227/21 Fase II—Corona-ômica BR MCTI Rede nacional de genomas, exoma e transcriptomade COVID-19 (JA). This work was supported by the National Council for Scientific and Technological Development (CNPq): Projects numbers 405547/2021–8 (JA) and 303857/2021-8 (JA). SP received a CAPES (The Coordination of Improvement of Higher Education Personnel - Brazil) fellowship grant Finance Code 001. VB received a CNPq fellowship grant no. 132611/2020-1.

## Conflict of interest

The authors declare that the research was conducted in the absence of any commercial or financial relationships that could be construed as a potential conflict of interest.

## Publisher’s note

All claims expressed in this article are solely those of the authors and do not necessarily represent those of their affiliated organizations, or those of the publisher, the editors and the reviewers. Any product that may be evaluated in this article, or claim that may be made by its manufacturer, is not guaranteed or endorsed by the publisher.

## Footnotes

^1^ https://artic.network/ncov-2019

^2^ https://artic.network/rampart

^3^ https://github.com/nanoporetech/qcat
