# Supplementary material for: Co-circulation of Chikungunya virus, Zika virus, and serotype 1 of Dengue virus in Western Bahia, Brazil
Source: Front Microbiol. 2023 Aug 23;14:1240860. doi: 10.3389/fmicb.2023.1240860 (PMC10482036; doi:10.3389/fmicb.2023.1240860)
Supplement: Supplementary file 2 [file Data_Sheet_2.zip › Supplementary Material 2.docx]

**Supplementary Material 2 -** Sequences used for designing DENV-1 primers.

| Strain Name | Virus Type | Gen Bank Accession | Collection Date | Host | Country |
| --- | --- | --- | --- | --- | --- |
| 21814/BR-PE/96 | Dengue virus 1 | JX669467 | 1996 | Human | Brazil |
| 41111/BR-PE/97 | Dengue virus 1 | JX669469 | 1997 | Human | Brazil |
| 40604/BR-PE/97 | Dengue virus 1 | JX669468 | 1997 | Human | Brazil |
| DENV1-2_BE_H_584526 | Dengue virus 1 | OK605754 | 1997 | Human | Brazil |
| 52082/BR-PE/98 | Dengue virus 1 | JX669470 | 1998 | Human | Brazil |
| 59049/BR-PE/99 | Dengue virus 1 | JX669471 | 1999 | Human | Brazil |
| DENV-1/BR/BID-V2374/2000 | Dengue virus 1 | FJ850070 | 2000 | Human | Brazil |
| DENV-1/BR/BID-V2375/2000 | Dengue virus 1 | FJ850071 | 2000 | Human | Brazil |
| 70523/BR-PE/00 | Dengue virus 1 | JX669472 | 2000 | Human | Brazil |
| SB 01057805 (DF02) | Dengue virus 1 | AB519681 | 2001 | Unknown | Brazil |
| DENV-1/BR/BID-V2378/2001 | Dengue virus 1 | FJ850073 | 2001 | Human | Brazil |
| 75861/BR-PE/01 | Dengue virus 1 | JX669474 | 2001 | Human | Brazil |
| 74488/BR-PE/01 | Dengue virus 1 | JX669473 | 2001 | Human | Brazil |
| DENV-1/BR/BID-V2381/2002 | Dengue virus 1 | FJ850075 | 2002 | Human | Brazil |
| 88463/BR-PE/02 | Dengue virus 1 | JX669475 | 2002 | Human | Brazil |
| DENV-1/BR/BID-V2384/2003 | Dengue virus 1 | FJ850077 | 2003 | Human | Brazil |
| DENV-1/BR/BID-V2389/2004 | Dengue virus 1 | FJ850081 | 2004 | Human | Brazil |
| DENV-1/BR/BID-V2392/2005 | Dengue virus 1 | FJ850084 | 2005 | Human | Brazil |
| DENV-1/BR/BID-V2395/2006 | Dengue virus 1 | FJ850087 | 2006 | Human | Brazil |
| DENV-1/BR/BID-V2398/2007 | Dengue virus 1 | FJ850090 | 2007 | Human | Brazil |
| DENV-1/BR/BID-V3490/2008 | Dengue virus 1 | GU131863 | 2008 | Human | Brazil |
| DENV-1/BR/BID-V2401/2008 | Dengue virus 1 | FJ850093 | 2008 | Human | Brazil |
| 13671/BR-PE/10 | Dengue virus 1 | JX669464 | 2010 | Human | Brazil |
| 13501/BR-PE/10 | Dengue virus 1 | JX669463 | 2010 | Human | Brazil |
| 9808/BR-PE/10 | Dengue virus 1 | JX669461 | 2010 | Human | Brazil |
| 14985/BR-PE/10 | Dengue virus 1 | JX669466 | 2010 | Human | Brazil |
| 13861/BR-PE/10 | Dengue virus 1 | JX669465 | 2010 | Human | Brazil |
| 12898/BR-PE/10 | Dengue virus 1 | JX669462 | 2010 | Human | Brazil |
| DENV1 BR/SJRP/17/2010 | Dengue virus 1 | KP188539 | 10/13/2010 | Human | Brazil |
| DENV1 BR/SJRP/287/2011 | Dengue virus 1 | KP188540 | 05/03/2011 | Human | Brazil |
| DENV1 BR/SJRP/354/2011 | Dengue virus 1 | KP188541 | 05/24/2011 | Human | Brazil |
| BR/SJRP/395/2011 | Dengue virus 1 | KP188542 | 06/08/2011 | Human | Brazil |
| DENV1 BR/SJRP/484/2012 | Dengue virus 1 | KP188543 | 02/13/2012 | Human | Brazil |
| DENV1 BR/SJRP/509/2012 | Dengue virus 1 | KP188544 | 03/16/2012 | Human | Brazil |
| DENV1 BR/SJRP/807/2013 | Dengue virus 1 | KP188547 | 01/05/2013 | Human | Brazil |
| BR/SJRP/885/2013 | Dengue virus 1 | KP188548 | 01/14/2013 | Human | Brazil |
| DENV1 BR/SJRP/778/2013 | Dengue virus 1 | KP188546 | 02/04/2013 | Human | Brazil |
| BR/SJRP/1107/2013 | Dengue virus 1 | KP188567 | 02/26/2013 | Human | Brazil |
| BR/SJRP/709/2013 | Dengue virus 1 | KP188545 | 10/25/2013 | Human | Brazil |
| BR/SJRP/2271/2014 | Dengue virus 1 | KP188568 | 02/10/2014 | Human | Brazil |
| USP-CB-53 | Dengue virus 1 | MW208043 | 11/11/2018 | Human | Brazil |
| USP-CB-54 | Dengue virus 1 | MW208044 | 11/18/2018 | Human | Brazil |
| USP-CB-111 | Dengue virus 1 | MW208056 | 06/05/2019 | Human | Brazil |
| DF01-HUB01021093 | Dengue virus 1 | FJ384655 | -N/A- | Human | Brazil |
