# Supplementary material for: Co-circulation of Chikungunya virus, Zika virus, and serotype 1 of Dengue virus in Western Bahia, Brazil
Source: Front Microbiol. 2023 Aug 23;14:1240860. doi: 10.3389/fmicb.2023.1240860 (PMC10482036; doi:10.3389/fmicb.2023.1240860)
Supplement: Supplementary file 4 [file Data_Sheet_4.zip › Supplementary Material 4.docx]

| RT-PCR result | Number of patients | Age (Mean±SD) | Female | Male |
| --- | --- | --- | --- | --- |
| DENV-1 | **32** | **19.93±16.85** | **15** | **17** |
| CHIKV | **45** | **26.53±15.33** | **25** | **20** |
| ZIKAV | **6** | **22.83±11.72** | **5** | **1** |
| NON-DETECTED | **15** | **24.86±14** | **7** | **8** |
| TOTAL | **98** | **-** | **52** | **46** |

Supplementary Material 4 – Demographic information of the study population according to the RT-PCR result.
